# Educational Attainment and Adolescent Foster Care Living Situations Among United States Women: A Cross-Sectional Analysis of the 2017-2019 National Survey of Family Growth

**DOI:** 10.7759/cureus.106282

**Published:** 2026-04-01

**Authors:** Aliyu A Tijani, Damilola Adekunle, Toyosi S Adedara, Modupe G Lawal, Emmanuel Abrefah, Adaobi Muorah, Benjamin O Akangbe

**Affiliations:** 1 Education, Texas Juvenile Justice Department, Austin, USA; 2 Social and Behavioral Health Science, Baylor University, Waco, USA; 3 Education, Baylor University, Waco, USA; 4 Environmental Health Sciences, University of Michigan, Ann Arbor, USA; 5 Public Health, Baylor University, Waco, USA; 6 Social Work, Baylor University, Waco, USA; 7 Nursing, Achievers University, Owo, NGA; 8 Public Health, Georgia State University, Atlanta, USA

**Keywords:** adolescent living arrangements, adverse childhood experiences (aces), educational achievement, educational attainment, educational attainment health disparities, foster care, national survey of family growth (nsfg), socioeconomic determinants, usa: united states of america

## Abstract

Children and adolescents in foster care face a higher risk of poor educational outcomes; however, quantitative studies using national data in the US are still limited. This study investigates the relationship between living situations at age 14 years and educational attainment in adulthood among women in the US. We conducted a cross-sectional secondary analysis of the 2017-2019 National Survey of Family Growth, focusing on women aged 25-49 years (N = 6,141). Living situations at age 14 years were categorized as (1) living with both biological or adoptive parents, (2) living with a biological mother and stepfather, or (3) living in other parental or non-parental situations (including foster care). Educational attainment was dichotomized as high (≥13 years) or low (<13 years). Multivariable logistic regression models examined associations between adolescent living situations and educational outcomes, adjusting for race, income, household size, and foster parent education. Among participants, 24.8% lived in foster care or non-parental situations at age 14 years. Higher educational attainment was significantly associated with living with both parents at age 14 years, higher household income, foster parent education, and fewer household members. Adjusted odds of higher education attainment were lower for those in foster care settings (adjusted odds ratio or AOR 0.77, 95% CI: 0.66-0.91) than those living with both parents. Foster care living situations in adolescence are independently associated with lower educational attainment in adulthood among US women. Targeted educational and social support interventions for foster youth are needed to address this disparity. Future longitudinal studies should validate these findings and explore underlying mechanisms.

## Introduction

Foster care in the US is designed as a temporary arrangement for children and adolescents who are unable to live with their biological families due to neglect, abuse, death, disability, substance use, incarceration, or when they are deemed to be unsafe in their biological home [1-3]. Over 368,000 children resided in the foster care system in the US in 2022 [4]. Of the children and youth in foster care, 110,230 were between 13 and 20 years of age, underscoring the significant representation of this age group within the foster care population [4]. Additionally, a recent report from the U.S. Department of Education showed that at any given time, approximately 400,000-435,000 children and youth are living in foster care nationwide, with a significant portion of this population comprised of adolescents facing transitions across home placements and schools [5]. While AFCARS (2022) reported 368,000 children in foster care, the U.S. Department of Education estimates a range of 400,000-435,000 at any given time; this discrepancy reflects differences in data sources, reporting periods, and administrative methodologies between child welfare and educational tracking systems.

Youth in foster care experience disproportionately high rates of educational disruption and instability compared with their peers. Frequent moves between foster homes often result in multiple school changes. Extant studies show that one-third of foster youth change schools five or more times before age 18 years, a factor strongly linked with gaps in academic progress and social continuity. In comparison to classmates who are not fostered, research has consistently demonstrated that these disruptions lead to lower academic achievement, persistent absenteeism, and an increased risk of grade retention and disciplinary actions [6-8].

Adolescent living situations, particularly those involving foster care or other non-parental arrangements, can profoundly shape life trajectories through mechanisms involving reduced educational support, economic instability, and limited access to academic resources [9]. Adoption and twin studies suggest that environmental factors such as caregiver education and household structure exert a significant influence on educational attainment, independent of genetic predisposition [10]. Prior qualitative studies have also highlighted the cumulative disadvantages foster youth face, including disrupted schooling, poor employment opportunities, frequent relocations, engagement in delinquent behavior, and exposure to additional adversities such as homelessness and substance abuse [11].

Adopted children from foster care typically range in age from one to five years. The likelihood of adopting older children from foster care is lower. A child’s prospects of being adopted are significantly reduced the longer they stay in foster care, even for as little as 12 to 18 months [5,12]. The history of trauma leading to foster care placement, combined with the vulnerability of foster care placement, contributes to the level of risk that youth experience when aging out of foster care. Beyond those currently in placement, a critical and often overlooked population is youth who age out of the foster care system, those who reach adulthood without being reunified with family or adopted, and those who face a distinct and compounded set of challenges. Youth in foster care are directly impacted by trauma, which increases their vulnerability to mental health problems as they age out of the system, such as signs of post-traumatic stress disorder, sadness, or anxiety [7]. Additionally, youth aging out of foster care have an increased risk of adverse outcomes, including physical and mental health alterations, unemployment, early pregnancy, substance use, limited access to education and health care, and lack of housing. These challenges are particularly acute for former foster youth who, having aged out of the system, must navigate the transition to adulthood, including post-secondary education and employment without the family support structures typically available to their peers [13]. Generally, most young people are unprepared to live on their own as soon as they turn 18. Independence without adult support and guidance is particularly difficult for children in foster care. It comes at a time when they are graduating high school and preparing to navigate higher education or start their careers [14].

A growing body of research underscores the complex interplay between living situation stability and educational outcomes for adolescents in foster care. Multiple studies show that frequent changes in schools and placements interfere with academic progress, social connections, and school continuity, all of which are important factors in academic success [14,15]. Disrupted school experiences are linked to poorer test scores, higher absenteeism rates, worse grade retention, and worse graduation outcomes when compared to peers who are not in care, according to a number of studies [14,16].

The relationship between living situations and educational outcomes is complex. Residential instability, school mobility, and placement changes undermine consistency in learning environments and access to stable adult support, both of which are well-established predictors of academic success. This structural background explains why it is impossible to understand the educational attainment of adolescents in foster care apart from their living conditions. Despite substantial qualitative and longitudinal research on the well-being of youth in foster care, quantitative analyses using recent nationally representative data remain scarce. Furthermore, few studies have specifically focused on how adolescent living arrangements correlate with adult educational attainment, particularly in the US context. This study aimed to address this gap by examining the association between adolescent living situations (at age 14 years) and educational attainment in adulthood, using multivariable logistic regression with adjustment for race, household income, household size, and caregiver education, applied to nationally representative data from the 2017-2019 National Survey of Family Growth (NSFG). Understanding these relationships is crucial for identifying modifiable risk factors and informing public health interventions aimed at improving life outcomes for this vulnerable population [17-22].

## Materials and methods

Study design and data source

This cross-sectional secondary analysis used data from the 2017-2019 NSFG, a nationally representative household survey conducted by the Centers for Disease Control and Prevention [23]. The NSFG collects detailed information on family life, fertility, and health behaviors of the civilian, non-institutionalized US population.

Study population

The analysis included female respondents aged 25 to 49 years (N = 6,141) who provided retrospective information about their living situation at age 14 years. This age range was selected to capture adult educational attainment outcomes while ensuring sufficient recall accuracy regarding adolescent living circumstances.

Inclusion and exclusion criteria

Inclusion criteria were female gender, age between 25 and 49 years, and complete data for key study variables. Respondents with missing data on living situation, educational attainment, income, race, household size, or caregiver education were excluded from multivariable analyses.

Key variables

Primary Outcome

Educational attainment was dichotomized as high (≥13 years of education) or low (<13 years), based on self-reported total years of schooling completed.

Primary Exposure

Living situation at age 14 years was categorized as (1) living with both biological or adoptive parents, (2) living with biological mother and stepfather, and (3) living in any other parental or non-parental situation (used as a proxy for foster care based on NSFG definitions and national foster care policy norms).

Covariates

The following covariates were included: race (White, Black, and other), household income (high vs. low), number of people in the household at age 14 years, educational attainment of foster parent or mother figure, and respondent’s current age.

Statistical analysis

Descriptive statistics were generated using univariate and bivariate procedures (PROC SURVEYFREQ and PROC SURVEYMEANS) in SAS version 9.4 (SAS Institute, Cary, NC), accounting for the complex survey design and sampling weights. Chi-square (χ²) tests evaluated bivariate associations between living situation and educational attainment; chi-square values and degrees of freedom are reported alongside p-values.

Multivariable logistic regression models (PROC SURVEYLOGISTIC) were used to estimate OR and 95% CI for the association between living situation at age 14 years and educational attainment, adjusting for covariates. Wald chi-square test statistics are reported alongside p-values for each predictor. Collinearity diagnostics and goodness-of-fit tests (Hosmer-Lemeshow) were performed to assess model stability. Statistical significance was set at p<0.05.

Ethics statement

The NSFG is a publicly available, de-identified dataset. This secondary analysis was exempt from Institutional Review Board review in accordance with US federal guidelines for research on publicly available data.

## Results

A total of 6,141 women aged 25 to 49 years were included in this study. The mean age of participants was 32.12 years (standard error = 0.22). Overall, 24.8% of the study population reported living in a foster care or other non-parental setting at age 14 years, while 65.7% lived with both biological or adoptive parents, and 9.5% lived with a biological mother and stepfather (Table 1).

**Table 1:** Weighted sociodemographic characteristics of female participants, NSFG 2017-2019 (N = 6,141) Data are presented as N (%) for categorical variables and mean ± SE for continuous variables. Statistical significance was set at p<0.05. The prevalence of high educational attainment (≥13 years) was 60.9%. Among participants who lived in foster care or other non-parental settings at age 14 years, only 39.1% achieved high educational attainment compared with 68.2% of those who lived with both parents (p<0.001). Footnote: Living situation categories are grouped to distinguish between non-foster care (both biological/adoptive parents, biological mother, and stepfather) and foster care/non-parental arrangements. The combined non-foster care subtotal is presented to enable direct comparison with foster care/non-parental living situations. Foster care/non-parental includes any living arrangement without a biological or adoptive parent present, consistent with NSFG classification. NSFG, National Survey of Family Growth.

Variable	N (%) or mean ± SE
Age (years)	32.12 (0.22)
Educational attainment (years)	13.73 (0.09)
High education (≥13 years)	3,740 (60.9%)
Low education (<13 years)	2,401 (39.1%)
Household income	
High	4,188 (68.2%)
Low	1,953 (31.8%)
Living situation at age 14 years	
Both biological/adoptive parents	4,035 (65.7%)
Biological mother + stepfather	583 (9.5%)
Foster care/other non-parental	1,523 (24.8%)
Race	
White	4,624 (75.3%)
Black	989 (16.1%)
Other	528 (8.6%)
Number of people in household (mean)	3.52 (0.05)
Foster parent education	
Less than high school	76 (1.23%)
High school graduate/GED	61 (0.99%)
Some college	47 (0.77%)
Bachelor's degree or higher	61 (1.00%)
No mother figure identified	11 (0.18%)

The prevalence of high educational attainment (≥13 years) was 60.9%. Among participants who lived in foster care or other non-parental settings at age 14 years, only 39.1% achieved high educational attainment compared with 68.2% of those who lived with both parents (p<0.001) (Table 1).

Bivariate analyses (Table 2) showed significant associations between educational attainment and race (p<0.001), household income (p<0.001), living situation at age 14 years (p<0.001), number of household members (p<0.001), and foster parent education (p<0.001).

**Table 2:** Weighted comparison of educational status by sociodemographic characteristics Data are presented as N (%) within each educational attainment group. Chi-square (χ²) tests were used to assess bivariate associations. Statistical significance was set at p<0.05. Bivariate analyses showed significant associations between educational attainment and race (p<0.001), household income (p<0.001), living situation at age 14 years (p<0.001), number of household members (p<0.001), and foster parent education (p<0.001).

Characteristic	High education, N (%)	Low education, N (%)	χ² (df), p-value
Living situation at age 14 years			χ²(2) = 405.63, p<0.001
Both biological/adoptive	2,551 (68.2%)	893 (37.2%)	
Mother + stepfather	355 (9.5%)	113 (4.7%)	
Foster care/other	928 (24.8%)	305 (12.7%)	
Household income			χ²(1) = 1148.33, p<0.001
High	2,551 (68.2%)	574 (23.9%)	
Low	509 (13.6%)	514 (21.4%)	
Race			χ²(2) = 53.78, p<0.001
White	2,816 (75.3%)	1,652 (68.8%)	
Black	602 (16.1%)	567 (23.6%)	
Other	322 (8.6%)	182 (7.6%)	
Foster parent education			χ²(3) = 142.51, p<0.001
Less than high school	337 (9.0%)	351 (14.6%)	
High school graduate/GED	583 (15.6%)	346 (14.4%)	
Some college	557 (14.9%)	204 (8.5%)	
Bachelor's degree or higher	546 (14.6%)	170 (7.1%)	

Unadjusted logistic regression (Table 3) indicated that compared to participants living with both parents at age 14 years, those in foster care or non-parental settings had significantly lower odds of high educational attainment (OR = 0.47, 95% CI: 0.39-0.57). Higher foster parent education, higher income, and smaller household size were also associated with increased odds of higher educational attainment.

**Table 3:** Unadjusted OR for high educational attainment Data are presented as OR (95% CI). Logistic regression was used to estimate ORs; Wald chi-square test statistics are reported. Statistical significance was set at p<0.05. Unadjusted logistic regression indicated that compared to participants living with both parents at age 14 years, those in foster care or non-parental settings had significantly lower odds of high educational attainment (OR = 0.47, 95% CI: 0.39-0.57). Higher foster parent education, higher income, and smaller household size were also associated with increased odds of higher educational attainment.

Predictor	OR (95% CI)	Wald χ²(1), p-value
Foster care/other vs. both parents	0.47 (0.39-0.57)	60.83, p<0.001
High household income	2.61 (2.22-3.07)	134.58, p<0.001
Foster parent education (per level)	1.89 (1.71-2.09)	154.63, p<0.001
Number of people in household (per additional person)	0.80 (0.75-0.85)	48.84, p<0.001
Race (Black vs. White)	0.59 (0.49-0.71)	31.10, p<0.001

In adjusted models (Table 4), after controlling for race, income, number of household members, caregiver education, and age, the odds of high educational attainment remained significantly lower for participants from foster care or other non-parental settings (adjusted odds ratio (AOR) = 0.77, 95% CI: 0.66-0.91). Higher household income (AOR = 2.14, 95% CI: 1.85-2.47) and greater foster parent education (AOR = 1.44 per education category increase, 95% CI: 1.32-1.58) were strong predictors of educational attainment. The number of people in the household and race also remained significant predictors.

**Table 4:** AORs for high educational attainment. Data are presented as AOR (95% CI). Multivariable logistic regression was used to estimate AORs, controlling for race, income, household size, caregiver education, and age; Wald chi-square test statistics are reported. Statistical significance was set at p<0.05. In adjusted models, after controlling for race, income, number of household members, caregiver education, and age, the odds of high educational attainment remained significantly lower for participants from foster care or other non-parental settings (AOR = 0.77, 95% CI: 0.66-0.91). Higher household income (AOR = 2.14, 95% CI: 1.85-2.47) and greater foster parent education (AOR = 1.44 per education category increase, 95% CI: 1.32-1.58) were strong predictors of educational attainment. The number of people in the household and race also remained significant predictors. AOR, adjusted odds ratio.

Predictor	AOR (95% CI)	Wald χ²(1), p-value
Foster care/other vs. both parents	0.77 (0.66-0.91)	10.17, p=0.001
High household income	2.14 (1.85-2.47)	106.47, p<0.001
Foster parent education (per level)	1.44 (1.32-1.58)	63.21, p<0.001
Number of people in household (per additional person)	0.90 (0.83-0.98)	6.18, p=0.013
Race (Black vs. White)	0.80 (0.68-0.95)	6.84, p=0.009

## Discussion

This study provides new, nationally representative evidence that adolescent living situations are significantly associated with educational attainment in adulthood among US women. Our findings demonstrate that individuals who lived in foster care or other non-parental settings at age 14 years were substantially less likely to achieve higher educational attainment by mid-adulthood compared with those raised by both biological or adoptive parents, even after adjusting for socioeconomic and demographic factors.

These results are consistent with prior US and international studies showing that foster care youth are at heightened risk for poor academic outcomes [14,16-18]. Foster care placements often disrupt schooling, social stability, and continuity of academic support, which can have long-lasting effects on educational trajectories [19]. Our findings reinforce these patterns using recent national survey data and contribute to addressing the documented scarcity of US-based quantitative analyses on this topic [9].

Importantly, this study highlights the significant role of caregiver education and household income in predicting adult educational attainment, consistent with previous behavioral genetic and socioeconomic studies [9,10]. Foster parent education may serve as a proxy for both material resources and value placed on academic achievement within the household environment. Similarly, higher household income during adolescence likely reflects broader access to educational resources and opportunities.

The finding that larger household size was associated with lower educational attainment aligns with theories of resource dilution, where children in larger households may receive fewer parental resources, time, and educational support per capita. This pattern has been previously observed in both general and vulnerable populations, including foster youth [20].

Interestingly, race remained an independent predictor of educational attainment, with Black female participants demonstrating lower odds of higher education compared with White female participants after controlling for other factors. This underscores the intersecting influence of race, socioeconomic status, and family structure on educational outcomes, reflecting broader structural inequalities well-documented in US education systems.

Limitations

Several limitations should be acknowledged. First, the cross-sectional, retrospective nature of this study prevents causal inference. While we found strong associations between adolescent living situation and educational attainment, longitudinal studies are needed to confirm these temporal relationships, also in line with findings by Cage [2]. Second, the operationalization of “foster care” relied on self-reported non-parental living arrangements without official foster care status verification, which may introduce misclassification bias. However, given US child welfare policies mandating care for unaccompanied minors, this assumption is reasonable though imperfect [1]. Third, educational attainment and childhood conditions were self-reported and subject to recall bias. Fourth, this analysis included only female respondents aged 25-49 years, which limits generalizability to males and younger adults. Lastly, unmeasured confounders such as childhood trauma, school quality, and mental health status may influence both living situation and educational outcomes and were not captured in the NSFG data. 

## Conclusions

Using nationally representative data from the 2017-2019 NSFG, this study found that adolescent living situation is a significant independent predictor of adult educational attainment among US women. Compared to those raised with both biological or adoptive parents, women who spent adolescence in foster care or other non-parental settings had significantly lower odds of achieving higher education, even after adjusting for race, household income, caregiver education, household size, and age (AOR = 0.77, 95% CI: 0.66-0.91). Household income (AOR = 2.14) and foster parent education (AOR = 1.44 per category increase) were the strongest positive predictors, underscoring how socioeconomic circumstances during formative years exert a lasting influence on educational trajectories. These findings carry direct implications for policy and public health practice. Targeted interventions are needed to strengthen educational outcomes for foster youth, including stable placement policies that minimize school disruptions, academic mentoring programs, post-secondary financial support, and expanded training and resources for foster caregivers. Because race, household income, and household composition remained significant predictors in adjusted models, addressing educational disparities among foster youth must be embedded within a broader structural commitment to equity. Education is a core social determinant of health, shaping employment, income, and long-term well-being, and equitable access must be treated as a national policy priority.

This study contributes new quantitative, nationally representative evidence to a literature that has historically relied on smaller or regional samples, extending understanding of how adolescent living arrangements shape adult educational outcomes. Its cross-sectional design limits causal inference, and future longitudinal research should examine mediating pathways, including school stability, mental health, and social support networks. Identifying the mechanisms that drive educational disadvantage among foster youth will be essential for developing precise, effective interventions to reduce persistent disparities in educational attainment.

## References

[1] Ahmann E (2017). Supporting youth aging out of foster care. Pediatr Nurs.

[2] Cage J (2018). Educational attainment for youth who were maltreated in adolescence: investigating the influence of maltreatment type and foster care placement. Child Abuse Negl.

[3] Courtney ME, Dworsky A, Brown A, Cary C, Love K, Vorhies V (2011). Midwest Evaluation of the Adult Functioning of Former Foster Youth: Outcomes at Age 26. https://www.chapinhall.org/wp-content/uploads/Midwest-Eval-Outcomes-at-Age-26.pdf.

[4] (2021). Education Outcomes of Washington Students in Foster Care. https://erdc.wa.gov/sites/default/files/2024-10/Education%20Outcomes%20of%20Washington%20Students%20in%20Foster%20Care%202021.pdf.

[5] U.S. Department of Education. (2026). U.S. Department of Education: Students in foster care. https://www.ed.gov/teaching-and-administration/supporting-students/special-populations/students-foster-care.

[6] Grattan RE, Tryon VL, Lara N, Gabrielian SE, Melnikow J, Niendam TA (2022). Risk and resilience factors for youth homelessness in Western countries: a systematic review. Psychiatr Serv.

[7] Greeno EJ, Fedina L, Lee BR, Farrell J, Harburger D (2019). Psychological well-being, risk, and resilience of youth in out-of-home care and former foster youth. J Child Adolesc Trauma.

[8] Hiles D, Moss D, Wright J (2013). Young people’s experience of social support during the process of leaving care: a review of the literature. Child Youth Serv Rev.

[9] Kelly V, Simmel C (2020). Current context of federal policy for teens involved in or transitioning out of foster care. Child Welfare.

[10] McGue M, Rustichini A, Iacono WG (2017). Cognitive, noncognitive, and family background contributions to college attainment: a behavioral genetic perspective. J Pers.

[11] Mekonnen R, Noonan K, Rubin D (2009). Achieving better health care outcomes for children in foster care. Pediatr Clin North Am.

[12] Nugent CN, Ugwu C, Jones J, Newburg-Rinn S, White T (2020). Demographic, health care, and fertility-related characteristics of adults aged 18-44 who have ever been in foster care: United States, 2011-2017. Natl Health Stat Rep.

[13] Pecora PJ (2012). Maximizing educational achievement of youth in foster care and alumni: factors associated with success. Child Youth Serv Rev.

[14] Pirog MA, Jung H, Lee D (2018). The changing face of teenage parenthood in the United States: evidence from NLSY79 and NLSY97. Child Youth Care Forum.

[15] Smeyne CN, Trott CD, Furst-Holloway S (2025). Fostering self-determination: a review and research agenda advancing self-determination theory as a unifying framework for youth well-being in foster care research. Child Adolesc Soc Work J.

[16] Somers CL, Goutman RL, Day A, Enright O, Crosby S, Taussig H (2020). Academic achievement among a sample of youth in foster care: the role of school connectedness. Psychol Sch.

[17] Stone KJ, Muentner L, Lundgren B (2025). School-based outcomes, post-graduation plans and adult support among youth who have been in foster care. Child Youth Serv Rev.

[18] (2026). The Annie E. Casey Foundation: Extended foster care explained. https://www.aecf.org/blog/extended-foster-care-explained.

[19] U.S. Department of Education: Students in foster care (2026). U.S. Department of Education: Students in foster care. https://www.ed.gov/teaching-and-administration/supporting-students/special-populations/students-foster-care/students-in-foster-care?utm_source=chatgpt.com.

[20] U.S. Department of Health and Human Services, Administration for Children and Families. (2026). U.S. Department of Health and Human Services: AFCARS report #30. https://www.acf.hhs.gov/cb/report/afcars-report-30.

[21] VanGraafeiland B, Foli KJ, Snethen JA (2024). Youth aging out of foster care: implications for nurse practitioners. J Nurse Pract.

[22] Vinnerljung B, Öman M, Gunnarson T (2005). Educational attainments of former child welfare clients: a Swedish national cohort study. Int J Soc Welfare.

[23] (2026). Centers for Disease Control and Prevention, National Center for Health Statistics: National survey of family growth. https://www.cdc.gov/nchs/nsfg/index.htm.

